# Improvised Long Test Lengths via Stitching Scale Bar Method: Performance Evaluation of Terrestrial Laser Scanners per ASTM E3125-17

**DOI:** 10.6028/jres.125.017

**Published:** 2020-05-28

**Authors:** Shendong Shi, Bala Muralikrishnan, Vincent Lee, Daniel Sawyer, Octavio Icasio-Hernández

**Affiliations:** 1 State Key Laboratory of Precision Measuring Technology and Instruments, Tianjin University, Tianjin 300072, People’s Republic of China; 2 National Institute of Standards and Technology, Gaithersburg, MD 20899, USA; 3 Centro Nacional de Metrología, km. 4.5 Carretera a Los Cués, Municipio El Marqués, Querétaro C.P. 76246, México

**Keywords:** length error, performance evaluation, stitching scale bar, terrestrial laser scanner

## Abstract

Periodic performance evaluation is a critical issue for ensuring the reliability of data from terrestrial laser scanners (TLSs). With the recent introduction of the ASTM E3125-17 standard, there now exist standardized test procedures for this purpose. Point-to-point length measurement is one test method described in that documentary standard. This test is typically performed using a long scale bar (typically 2 m or longer) with spherical targets mounted on both ends. Long scale bars can become unwieldy and vary in length due to gravity loading, fixture forces, and environmental changes. In this paper, we propose a stitching scale bar (SSB) method in which a short scale bar (approximately 1 m or smaller) can provide a spatial length reference several times its length. The clear advantages of a short scale bar are that it can be calibrated in a laboratory and has potential long-term stability. An essential requirement when stitching a short scale bar is that the systematic errors in TLSs do not change significantly over short distances. We describe this requirement in this paper from both theoretical and experimental perspectives. Based on this SSB method, we evaluate the performance of a TLS according to the ASTM E3125-17 standard by stitching a 1.15 m scale bar to form a 2.3 m reference length. For comparison, a single 2.3 m scale bar is also employed for direct measurements without stitching. Experimental results show a maximum deviation of 0.072 mm in length errors between the two approaches, which is an order of magnitude smaller than typical accuracy specifications for TLSs.

## 1 Introduction

Terrestrial laser scanners (TLSs) are three-dimensional (3D) imaging systems that are widely used in large-volume measurements (at length scales of several meters to tens of meters). With the advantages of noncontact measurement, high point density, high data rate, and accuracy, they play an increasingly important role in applications such as deformation monitoring in civil engineering and geodesy [1–5], historical preservation and archiving [6–8], reverse engineering [9–10], and agricultural tasks [11–12].

To ensure the integrity of data obtained from a TLS, it is important to implement test procedures and assess their performance periodically. There currently exist two published documentary standards covering methods and testing procedures for evaluating the performance of TLSs: ASTM E2938-15 and ASTM E3125-17. The first standard is limited to evaluation along the ranging direction. The second standard is more comprehensive and covers evaluation within the entire working volume of TLSs, describing several types of point-to-point length tests, including symmetric tests, asymmetric tests, inside tests, relative-range tests, and user-specified tests.

**Fig. 1:**
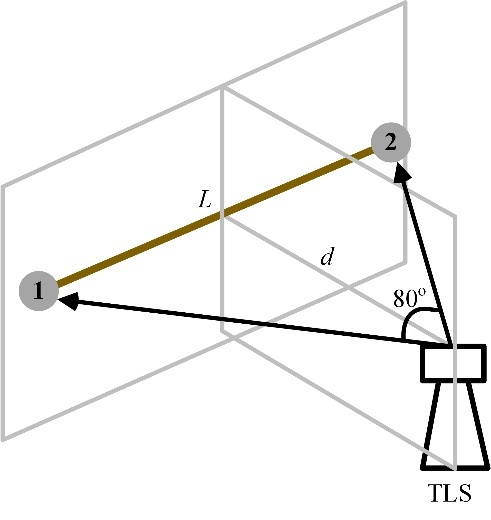
The angular sweep of the TLS in symmetric horizontal tests.

In this paper, we only discuss the symmetric length tests because they pose a special challenge. Nevertheless, all the length tests are within the scope of this paper. According to the requirements in ASTM E3125-17, the angular sweep between targets at the ends of a symmetric reference length must be at least 80°. As shown in Fig. 1, for a scale bar horizontally placed at the same height as the TLS, the minimum scale bar length L and the distance d from the scale bar to the TLS follow the linear relationship in Eq. (1):

L=2dtan40 (1)

At a distance of 2 m, the minimum length of the scale bar is therefore 3.4 m. The construction and calibration of such a long scale bar create challenges for manufacturers, as well as for customers. Because of the structural bending under gravity loading and fixture forces, the length of a long scale bar can undergo significant changes in dimension at different orientations [13]. The length of a scale bar made from carbon fiber can also change as a function of time due to changes in humidity. Hudlemeyer *et al*. [14] used a long scale bar for field checking of laser trackers, but in that case, the instrument under test was itself used to calibrate the scale bar *in situ*. TLSs, on the other hand, do not have equally accurate ranging capability, and, therefore, the *in situ* calibration of a long scale bar using a TLS would not yield acceptably small uncertainties for performance evaluation purposes. Icasio-Hernández *et al.* [15] proposed an overlapping method, where they used a short step gauge to evaluate the performance of a coordinate measurement machine (CMM) with long range by overlapping several gauging elements from one position to the next. Lee and Sawyer [16] proposed a simpler realization of Icasio-Hernández’s work, where a long reference length was constructed by overlapping just one gauging element. They used a short scale bar and simply repositioned it serially to build a long reference length. They demonstrated the application of this technique for interim tests of laser trackers.

We extend the work of Lee and Sawyer in the context of performance evaluation of a TLS by stitching a short scale bar to obtain the equivalent function of a long scale bar. This method requires that the systematic errors in the TLS do not vary significantly over short distances when compared to the measurement repeatability. We demonstrate the validity of this requirement through model-based theory analysis and experiments.

The paper is organized as follows. We introduce the stitching scale bar method (SSB method) and discuss the previously mentioned instrument requirements in Sec.2. We discuss a validation experiment of the SSB method in Sec. 3. In Sec. 4, we demonstrate the application of this method to realize the symmetric length tests according to the ASTM E3125-17 standard. The length tests are first performed by stitching two segments of a 1.15 m scale bar together, and then they are performed again using a 2.3 m scale bar for comparison purposes. In Sec. 5, we discuss the uncertainty in the length of the stitched long scale bar. Conclusions are presented in Sec. 6.

## 2 SSB Method Overview and Requirements

### 2.1 Method Overview

Suppose we have a scale bar constructed with two spheres at each end, as shown in Fig. 2(a). Let the reference length of the scale bar be $L_{ref}$. The scale bar is first placed at position 1, and its length as measured by the TLS is $L_{1}$. The scale bar is then moved to position 2. It is not practically feasible for sphere 1 at position 2 to identically overlap sphere 2 at position 1. We discuss this overlap requirement later but simply note that the overlap is performed to the best ability of the operator and typically to within a few

**Figure:**
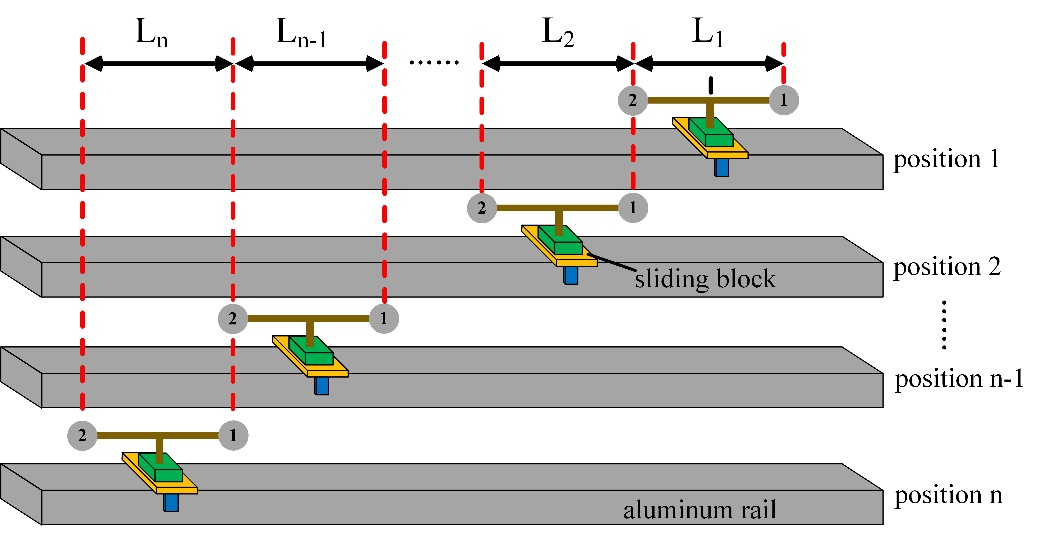
(a)

**Figure:**
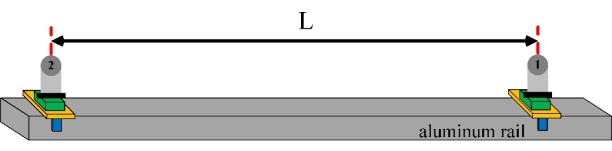
(b)

**Fig. 2:** (a) Schematic diagram of the SSB method and (b) equivalent long scale bar of length L achieved by the SSB method in (a).

millimeters. Let the length of the scale bar at position 2 recorded by the TLS be $L_{2}$. This process may be repeated n times. The point-to-point length error for each measurement at position i is expressed as:

$e_{i}=L_{i}-L_{ref}$ (2)

where i is the position index of the scale bar, and $e_{i}$ is the length error of the scale bar at position i.

Then, the length error e over the length L in Fig. 2(b), *i.e*., the distance between sphere 1 at position 1 and sphere 2 at position n, is formulated as the superposition of $e_{i}$:

$e=\sum_{i=1}^{n}e_{i}$ (3)

In this SSB method, the sum of length errors is used in place of the length error we would have obtained if we had measured the length L in Fig. 2(b). This method is only valid under a key requirement that the systematic errors in the TLS do not vary significantly over short distances when compared with the measurement repeatability. We discuss this requirement in the following subsections and show both model-based analysis and experimental data establishing that this condition is met.

### 2.2 Model-Based Systematic Error in TLSs

**Fig. 3:**
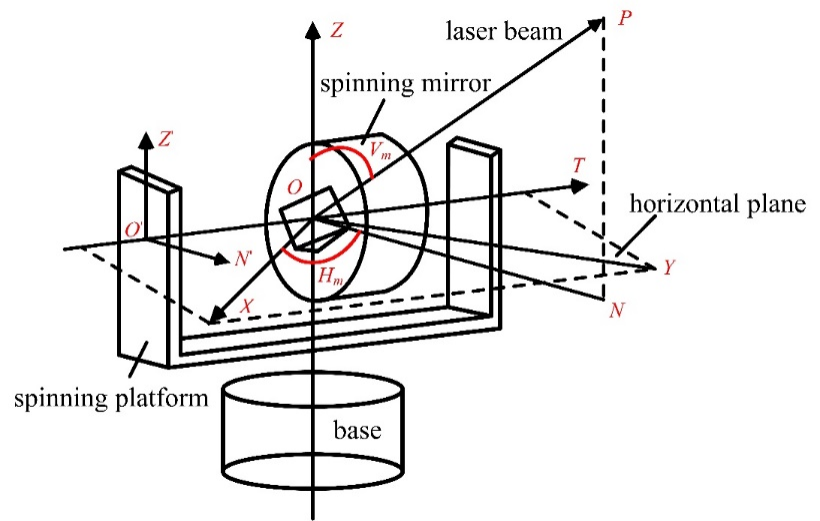
Coordinate system definition for an ideal TLS.

The TLS is a spherical coordinate measurement system consisting of length and angle measurement modules. Muralikrishnan *et al*. [17] proposed a geometric error model for the TLS shown in Fig. 3, and they discussed the individual error contributions (*i.e*., offsets, tilts, and eccentricities) in the optical and mechanical assembly.

Here, the model-based corrections to the measured range and the vertical and horizontal angles are simply listed in Eqs. (4), (5), and (6):

$∆R_{m}=k\left(x_{2}sinV_{m}\right)+x_{10}$ (4)

$∆H_{m}=k\left[\frac{x_{1z}}{R_{m}tanV_{m}}+\frac{x_{3}}{R_{m}sinV_{m}}+\frac{x_{5z}}{tanV_{m}}+\frac{2x_{6}}{sinV_{m}}-\frac{x_{7}}{tanV_{m}}-x_{8x}sinH_{m}+x_{8y}cosH_{m}\right]+\frac{x_{1n}}{R_{m}}+x_{5n}+$

$x_{11a}cos2H_{m}+x_{11b}sin2H_{m}$ (5)

$∆V_{m}=k\left[\frac{x_{1n}cosV_{m}}{R_{m}}+\frac{x_{2}cosV_{m}}{R_{m}}+x_{4}+x_{5n}cosV_{m}+x_{9n}cosV_{m}\right]-\frac{x_{1z}sinV_{m}}{R_{m}}-x_{5z}sinV_{m}-x_{9z}sinV_{m}+$

$x_{12a}cos2V_{m}+x_{12b}sin2V_{m}$ (6)

where:

$R_{m}$, $H_{m}$, and $V_{m}$ are the measured range, horizontal angle, and vertical angle, respectively;

${∆R}_{m}$, ${∆H}_{m}$, and $∆V_{m}$ are the corrections applied to the measured range, horizontal angle, and vertical

angle, respectively;

k is 1 for the front-face measurement and −1 for the back-face measurement;

$x_{1n}$ and $x_{1z}$ are beam offset along the N and Z axes, respectively;

$x_{2}$ is the transit offset;

$x_{3}$ is the mirror offset;

$x_{4}$ is the vertical index offset;

$x_{5n}$ and $x_{5z}$ are beam tilts along the N and Z axes, respectively;

$x_{6}$ is the mirror tilt;

$x_{7}$ is the transit tilt;

$x_{8x}$ and $x_{8y}$ are horizontal angle encoder eccentricity along the X and Y axes, respectively;

$x_{9n}$ and $x_{9z}$ are vertical angle encoder eccentricity along the N and Z axes, respectively;

$x_{10}$ is the zero offset or “bird-bath” error;

$x_{11a}$ and $x_{11b}$ are second-order scale errors in the horizontal angle encoder; and

$x_{12a}$ and $x_{12b}$ are second-order scale errors in the vertical angle encoder.

The corrections above show that the model terms are either trigonometric functions of horizontal or vertical angles or constant values (such as vertical index offset and zero offset). Therefore, the systematic errors caused by geometry misalignments in any direction do not vary significantly over short distances (the gap between overlapped spheres, *i.e*., the gap between sphere 2 in position *n* − 1 and sphere 1 in position *n* in Fig. 2[a]), *i.e.*, a few millimeters.

As an example, consider a target *A* located 5 m from the TLS and at a vertical angle of 45°. Let the beam offset term $x_{1n}$ be 0.01 mm. Then, the magnitude of the error along the *Z* direction is $R∆V_{m}$ = $x_{1n}cosV_{m}$ = 0.007 mm. Consider another target *B* located 5 mm above target *A*; the vertical angle to this target is 45.04°. Then, the error along the *Z* direction for target B is also 0.007 mm, which is negligibly similar (< 1 μm) to the error for target *A*. This example shows that the systematic errors in TLSs do not change significantly over short distances.

### 2.3 Measurement Repeatability of TLSs

The previous subsection establishes that the TLS errors are not expected to vary significantly over short distances. In this section, we discuss the experimental results quantifying the repeatability of the TLS under study, while in the next section, we discuss the experimental results showing that the systematic errors do not change significantly over short distances when compared with the repeatability.

The repeatability experiment was performed as follows. The aluminum sphere with a dull gray surface finish and a nominal diameter of 100 mm (we call it “single sphere” in the following context) in Fig. 4 was placed approximately 3 m from the TLS under test and measured 10 times. The point cloud corresponding to the sphere was extracted from the scan data with a cone-cylinder segmentation method, and the center coordinates were determined with an orthogonal nonlinear least-squares constrained-radius fitting [18]. The standard deviations of 10 measurements are reported in Table 1. The standard deviations of vertical and horizontal angles were multiplied by the average range of 10 measurements to obtain results in units of millimeters. We note that such repeatability measurements were performed at different measurement conditions; Table 1 is a representative example of values typically encountered. In Table 1, $\sigma_{R}$, $\sigma_{V}$, and $\sigma_{H}$ are one standard deviation of range, vertical angle, and horizontal angle, respectively, and R is the average range of 10 measurements.

**Figure:**
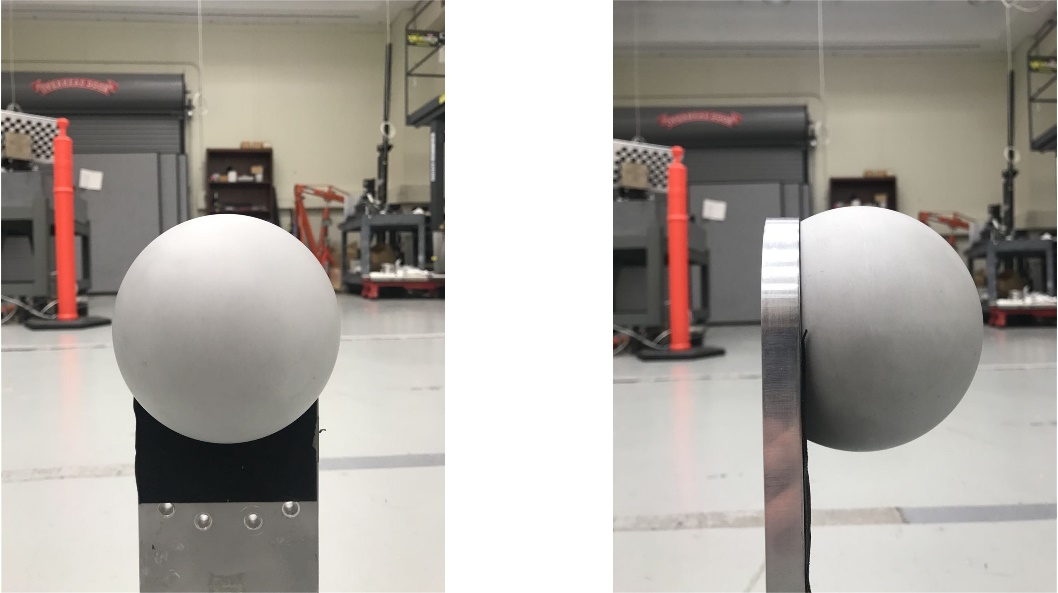
(a) (b)

**Fig. 4:** The single sphere used in repeatability experiments: (a) front view and (b) side view.

**Table 1:** Ranging axis and angle axes standard deviations for a single sphere 3 m from the TLS.

	**Standard Deviation (mm)**
$\sigma_{R}$	0.017
$R{∙\sigma }_{V}$	0.023
$R∙\sigma_{H}$	0.009

### 2.4 Systematic Error Evaluation over Short Distances

**Fig. 5:**
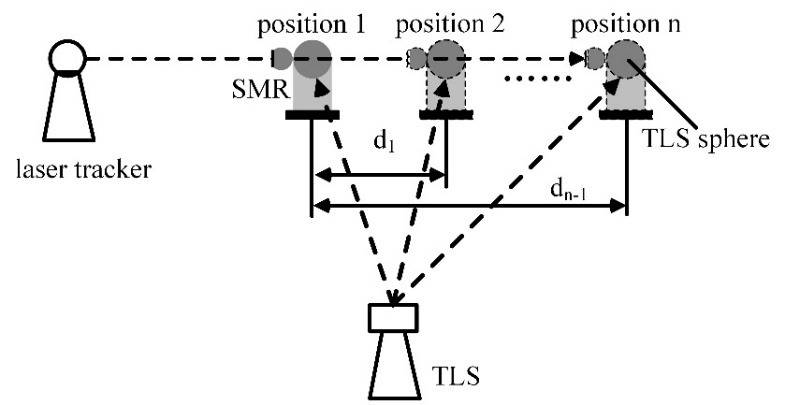
Experimental process of length error evaluation over short distances. SMR = spherically mounted retroreflector.

In this section, we discuss an experiment demonstrating that the TLS systematic errors do not vary significantly over short distances, *i.e*., a few millimeters. The experimental process is shown in Fig. 5.

A single sphere is mounted on the platform of a linear stage. A spherically mounted retroreflector (SMR) nest is also placed at the same height as the center of the sphere to minimize the Abbe offset. A laser tracker is used as the reference system. The linear stage is oriented so that the movement direction of the sphere is along the ranging direction of the laser tracker. The TLS is placed about 1.5 m from the sphere. The sphere is moved in steps of 1 mm from position 1 to position n. Position 1 is used as the reference position for calculating distances. By comparing the distances between positions i (i = 2 to n) and position 1 for the laser tracker and the TLS, we can understand the TLS length error distribution over short distances. Note that the errors along the movement direction of the sphere have a dominant effect in the SSB method. Thus, we fit a line using each set of data from the TLS and the laser tracker, and then we calculated the length errors along the movement direction.

**Fig. 6:**
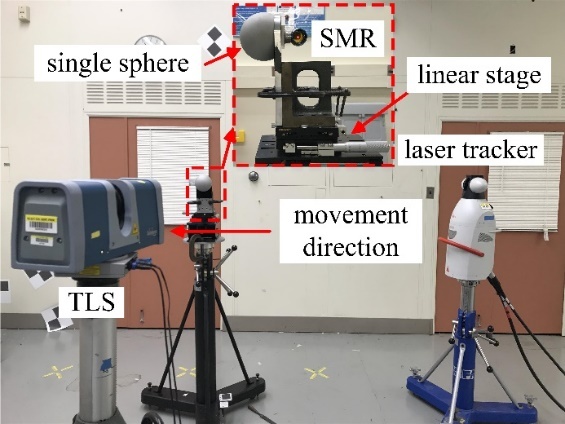
Experimental setup for systematic error evaluation over short distances.

**Fig. 7:**
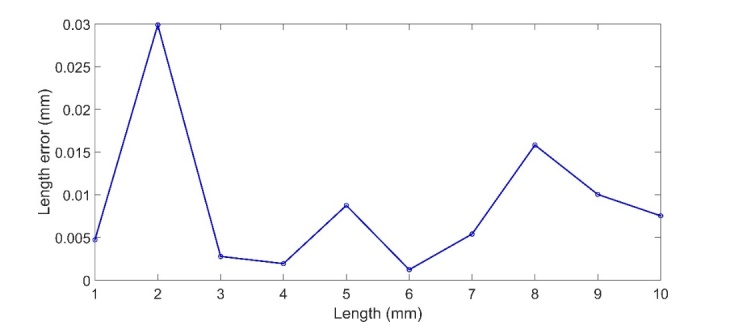
Length errors along the movement direction over a distance of 10 mm.

In this experiment, the sphere was moved to 10 different positions (positions 2–11), establishing 10 reference distances ranging from 1 mm to 10 mm. Two sets of 3D coordinates were acquired, one set from the measurements of the SMR by the laser tracker and the other set from the measurements of the single sphere by the TLS. The distances from the two systems were compared to determine the length errors of the TLS. The experimental setup and results are shown in Figs. 6 and 7, respectively.

As shown in Fig. 7, for all point pairs, the maximum deviation is about 30 μm when compared with the laser tracker. These errors are comparable to the repeatability of the TLS in Table 1. In other words, when we measure one reference length L and another length L+d (where d ranges from −5 mm to 5 mm) using the TLS, the difference in the length errors between the two measurements is within the TLS repeatability.

The model-based theory analysis, along with the two experiments above, clearly validates the requirement on TLS systematic errors and indicates that the SSB method is feasible for TLS performance evaluation.

## 3 Experimental Verification of the SSB Method

In Sec. 2, we discussed and verified the requirement of the SSB method through model-based analysis and experiments. To further test this method, we designed the following experiment. A scale bar of 500 mm nominal length with a single 100 mm diameter sphere at each end was used. The reference length $L_{ref}$ of the scale bar was measured on a CMM. The scale bar was first placed at position 1 on a long aluminum rail, as shown in Fig. 8(a). The TLS measured the center coordinates of the two spheres: $\left(x_{1},y_{1}{,z}_{1}\right)$ and $\left(x_{2},y_{2}{,z}_{2}\right)$. The length error of the scale bar at position 1 was then described as $e_{1}$ in Eq. (7):

$e_{1}=\sqrt{{\left(x_{1}-x_{2}\right)}^{2}+{\left(y_{1}-y_{2}\right)}^{2}+{\left(z_{1}-z_{2}\right)}^{2}}-L_{ref}$ (7)

The scale bar was then moved to position 2, where sphere 1 overlapped nominally with sphere 2 at position 1, and a new error $e_{2}$ was calculated. This was repeated for n positions. Then, the total error $e_{stitching}$ for a length L (from sphere 1 at position 1 to sphere 2 at position n) can be derived from Eq. (3).

To determine the validity of the length error obtained through the stitching process, the length error without stitching was measured as follows. A single sphere and an SMR were mounted at the same height. The SMR was close to the sphere to the fullest extent possible, as shown in Fig. 8(b). A laser tracker was placed in line to establish the reference length. The sphere was first measured at position 1 (overlapping with sphere 1 of the 500 mm scale bar at position 1) using the TLS, and the SMR position was measured by the laser tracker. The sphere was then moved to position 2 (overlapping with sphere 2 of the 500 mm scale bar at position n), and both the TLS and the laser tracker recorded the centers again. Let the distance between position 1 and position 2 of the single sphere as measured by the TLS be $L_{S}$ and the same distance measured by the laser tracker be $L_{T}$. The difference between $L_{S}$ and $L_{T}$ is the error in measuring a simulated long scale bar without stitching:

$e_{long}=L_{S}-L_{T}$ (8)

Thus, the deviation due to the SSB method is described in Eq. (9):

$e_{SSB}=e_{stitching}-e_{long}$ (9)

In this experiment, a scale bar with a calibrated length of 499.974 mm was used, and it was measured at four positions. Thus, an equivalent length of 2 m was constructed. Experimental results are shown in Table 2. When a 500 mm short scale bar is used to cover a length of 2 m, the deviation due to the SSB method is 0.025 mm, which is close to the measurement repeatability of the TLS in Table 1. Therefore, the SSB method is a potential option for the performance evaluation of TLSs.

**Figure:**
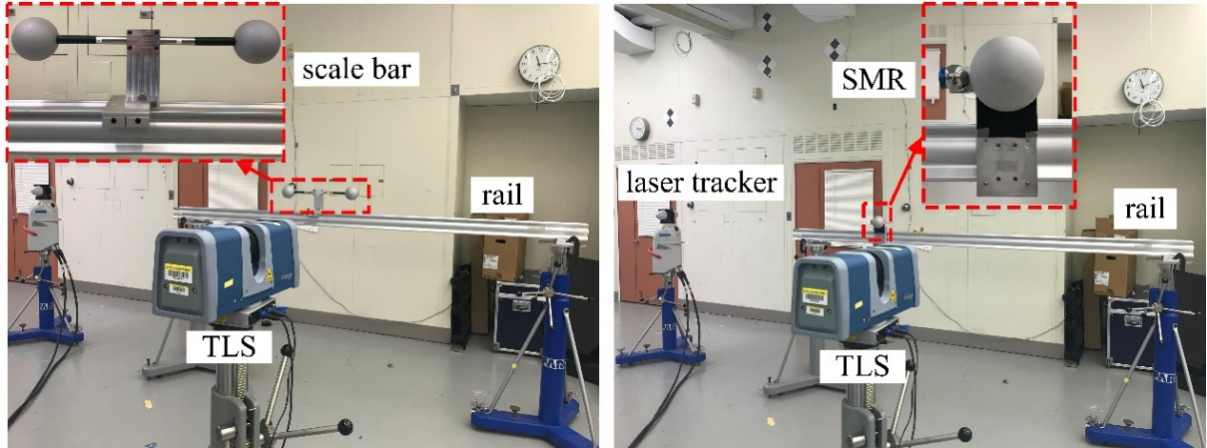
(a) (b)

**Fig. 8:** Experimental setup of (a) the SSB method and (b) the single sphere method without stitching.

**Table 2:** The length error of the SSB method when compared with laser tracker.

**SSB Method (mm)**					**Single Sphere Method (mm)**	**Deviation (mm)**
$e_{1}$	$e_{2}$	$e_{3}$	$e_{4}$	$e_{stitching}$	$e_{long}$	$e_{SSB}$
−0.027	0.008	0.034	0.035	0.050	0.025	0.025

## 4 Performance Evaluation of TLSs per ASTM E3125-17 with the SSB Method

In Sec. 3, we described an experiment validating the SSB method for generating a long reference length. In this section, we will discuss the performance evaluation of a TLS according to the ASTM E3125-17 standard with this method. As mentioned in Sec. 1, there are different types of length tests in the ASTM E3125-17 standard. We only discuss the realization of the symmetric length tests in this section, but the SSB method is applicable to all length tests.

**Fig. 9:**
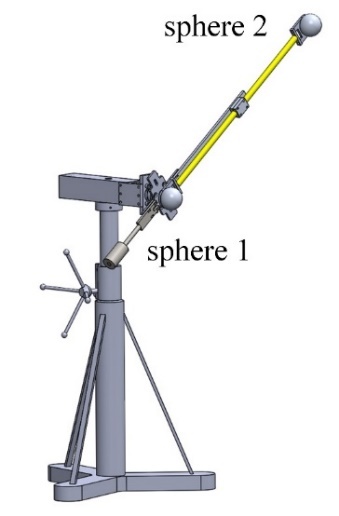
A 1.15 m short scale bar located on a tripod.

To realize the symmetrical length tests in the ASTM E3125-17 standard, we designed a 1.15 m short scale bar that was mounted on an arm with a rotational degree of freedom, as shown in Fig. 9. The scale bar was made out of an Invar tube and had a single sphere as the target at each end. These spheres were hollowed out to reduce their weights. The length of this scale bar was measured on a CMM in the horizontal orientation and later again after flipping the scale bar by 180° about its neutral axis; the length of the scale bar varied slightly with different orientations to gravity. The center of sphere 1 was nominally on the rotation axis, so that we could stitch the 1.15 m scale bar to form a 2.3 m reference length when rotating the scale bar in the vertical plane by approximately 180°.

To demonstrate the suitability of this scale bar to realize the symmetric length tests, we performed the following experiment. We first used the 1.15 m scale bar for symmetric length tests according to the ASTM E3125-17 standard. The center of the TLS was at the same height as the rotation axis of the scale bar. The TLS was 1.3 m from the scale bar, so that the angular sweep was more than 80°. We rotated the scale bar to four orientations: symmetric horizontal, symmetric vertical, symmetric left diagonal, and symmetric right diagonal in the view of the TLS. At each orientation, the two spheres of the scale bar were first measured at position 1. Then, we rotated the scale bar by 180° to position 2 and measured the coordinates of the two spheres again. The sum of length errors for the two measurements was taken as the equivalent length error while measuring a scale bar with double the length, *i.e*. 2.3 m. The experimental procedure is shown in Fig. 10.

**Figure:**
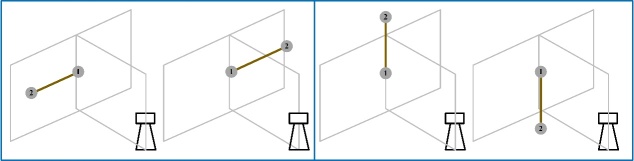
(a) (b)

**Figure:**
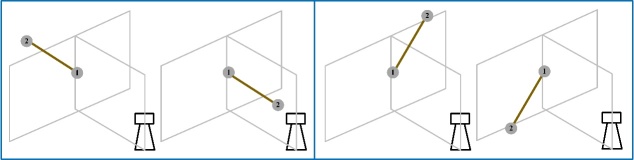
(c) (d)

**Fig. 10:** Experimental process of performance evaluation of a TLS using the SSB method: (a) horizontal orientation, (b) vertical orientation, (c) left diagonal orientation, and (d) right diagonal orientation.

The SSB method was validated by measuring the four symmetric lengths using a single 2.3 m scale bar. This scale bar was made of carbon fiber and had a single sphere mounted at each end. The two spheres were hollow, with a nest centrally located inside that allowed a 38.1 mm (1.5 inch) SMR to be seated at the center of the sphere, as shown in Fig. 11. The reference length of the 2.3 m scale bar was measured using a laser tracker. The measurement process is described next.

**Figure:**
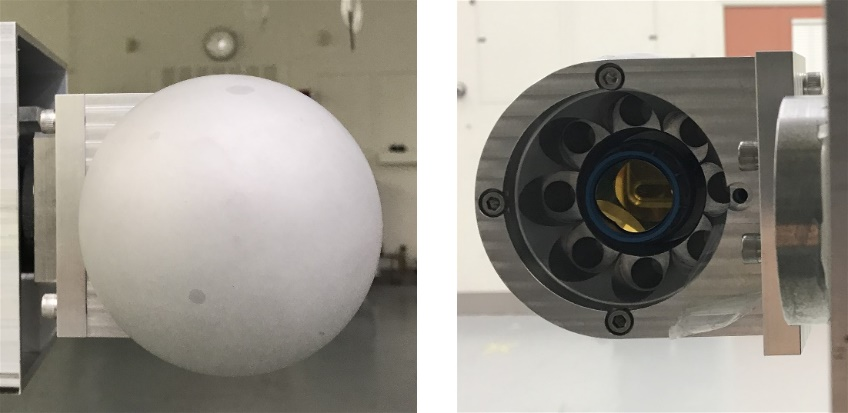
(a) (b)

**Fig. 11:** Single sphere with an SMR nest inside: (a) front view for TLS measurements and (b) back view for laser tracker measurements.

We rotated the scale bar to the horizontal orientation prior to measurements. The laser tracker was located behind the scale bar for line-of-sight access to the SMRs. The laser tracker was located at the same height as the scale bar. The measurement procedure using the laser tracker was described by Wang *et al*. [19], and it is referred to as the four-orientation and two-face method. As the name implies, the length is measured from four orientations of the laser tracker, where each orientation is rotated by 90° about the vertical axis from the previous orientation. At the first orientation of the laser tracker, we measured each SMR in the front and back face, respectively. We averaged the front-face and back-face coordinates for each SMR and calculated the length. Then, we rotated the laser tracker by 90° and repeated the process. We repeated this process two more times to obtain four lengths, one from each orientation. We averaged the four lengths to determine the final length of the scale bar. It has been shown by Wang *et al.* that this method provides reference lengths that are within ±10 μm (95% confidence intervals) of the length measured by line-of-sight interferometry.

After the measurement of the reference length, the 2.3 m scale bar was used for performance evaluation of the TLS. As noted earlier, only the symmetrical length tests described in the ASTM E3125-17 standard were performed. We adjusted the position and orientation of the TLS so that the encoder readings at each orientation when measuring the sphere away from the rotation axis of the short scale bar (sphere 2 in Fig. 9) at positions 1 and 2 of each orientation were the same as the readings when measuring the two spheres of the long scale bar. The experimental setup and results are shown in Fig. 12 and Table 3.

As shown in Table 3, the length errors obtained by the SSB method agree with the errors obtained using a long scale bar to approximately 0.07 mm. These errors are substantially smaller than typical accuracy specifications (range specification of 1 mm and angle specification of 30") of the TLS under test. We do not attempt to explain the observed behavior of the errors in Table 3 based on the error model parameters because we do not have enough information to make an educated guess. Only four length measurements were performed, whereas there are 18 parameters in the model. There may be many linear combinations of the model parameters that yield the observed errors.

**Figure:**
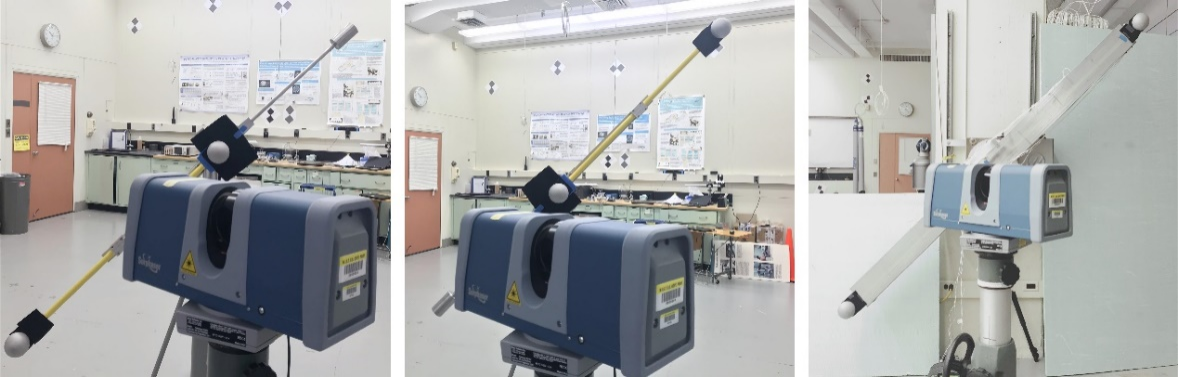
(a) (b) (c)

**Fig. 12:** (a) A 1.15 m scale bar at position 1 in the symmetric right diagonal orientation, (b) a 1.15 m scale bar at position 2 in the symmetric right diagonal orientation, and (c) a 2.3 m scale bar in the symmetric right diagonal orientation.

**Table 3:** Length errors between the SSB method and long scale bar method for TLS symmetric tests.

	**SSB Method (mm)**	**Long Scale Bar Method (mm)**	**Deviation (mm)**
**Horizontal**	−0.485	−0.446	−0.039
**Vertical**	−1.760	−1.832	0.072
**Left diagonal**	−0.251	−0.286	0.035
**Right diagonal**	−1.683	−1.618	−0.065

## 5 Uncertainty Analysis in the Stitched Scale Bar Length

When evaluating the performance of a TLS, we require the length of the scale bar at the instant in time at which the TLS performs the measurement. Thus, in this study, any change in the length of the scale bar from the time it was measured on a CMM to the time of its eventual use for TLS performance evaluation must be accounted for in the uncertainty of the scale bar length. The contributors to the uncertainty include uncertainty in the length as determined by the CMM, the effect of changes in temperature from the time it was measured on the CMM to the time of use for performance evaluation of the TLS, and the effects of mounting, gravity, and orientation. In Table 4, we first provide the uncertainty values for each component in the case of a 1.15 m scale bar when performing a single measurement, *i.e*., no stitching. We then discuss the case where we extend the reference length by the SSB method.

**Table 4:** Uncertainty contributions of each component.

**Uncertainty source**	Value (μm)
CMM measurement uncertainty $u_{c}$	0.2
Scale bar bending effects $u_{B}$	3.8
Temperature effects $u_{T}$	0.4
Combined standard uncertainty U	3.8
Expanded uncertainty **$U_{E}\left(k=2\right)$**	7.6

(1) CMM measurement uncertainty $u_{c}$: This is the uncertainty in the length of the scale bar measured using a CMM and corrected to a temperature of $T_{0}$ = 20 °C. The stylus used was 8 mm in diameter with a spherical tip made from silicon nitride and configured in a “L shape.” While remaining in its fixture, the scale bar was measured on a CMM using 49 measurement points evenly distributed across the same hemispheres measured by the laser scanner. These measurement points were evaluated using a least-square fit to calculate the sphericity, diameter, and location of the center. The length of this scale bar was determined by calculating the distance between the centers of these two spheres. This measurement was repeated 10 times to obtain a standard deviation of 0.15 μm. To compensate for systematic errors, a step gauge with a calibration uncertainty of 0.11 μm was measured. Summing these values in quadrature yielded a standard uncertainty of 0.2 μm.

(2) Scale bar bending effects $u_{B}$: Our current scale bar prototype is a modification of the short scale bar proposed by Lee and Sawyer [16], which was designed for laser trackers. The laser tracker scale bar accommodated a 38.1 mm (1.5 inch) SMR at each end, while our scale bar consisted of two heavier 100 mm diameter aluminum spheres. The length of the scale bar was measured on a CMM at two different orientations, exhibiting a difference in the measured length of 13 μm. We took the mean as the reference value and used the difference as bounds of a rectangular distribution to calculate the uncertainty due to bending effects.

(3) Temperature effects $u_{T}$: If the temperature at the time of testing is T (different from calibration temperature $T_{0}$), the length of the scale bar can be corrected for expansion or contraction. In our case, the reference length was measured on a CMM at 20 ℃, while the temperature of the room in which the scale bar was measured by the TLS was (20±0.5) ℃. Assuming 0.5 ℃ as the bounds of a rectangular distribution and 1.2μm/m/°C as the thermal coefficient of expansion of Invar, we estimated the standard uncertainty in the length of the scale bar due to temperature effects to be 1.15 m × 1.2 μm/m/°C × 0.5 ℃/$\sqrt{3}$ = 0.4 μm.

(4) Mounting effects: The scale bar was held in the same mounting mechanism during the measurement on the CMM and its subsequent use for TLS performance evaluation. Therefore, mounting did not contribute to the uncertainty of its length.

Summing these terms in quadrature, the standard uncertainty U in the length of the 1.15 m scale bar is 3.8 μm; the expanded uncertainty with a confidence interval of 95% (coverage factor k = 2) is $U_{E}$ = 7.6 μm.

When two 1.15 m long lengths are stitched together to form a 2.3 m reference length, any error in the calibration of the scale bar or any change in the length due to temperature will double because the same scale bar is measured twice [15]. The same is not necessarily true for the bending component. However, as a conservative estimation of the uncertainty due to this source, this component was doubled as well. Thus, the overall expanded uncertainty (k = 2) for the 2.3 m reference length is 15.2 μm. Compared with the general accuracy specifications of the TLS under test, the uncertainty in the length of the stitched scale bar has a much smaller order of magnitude, which validates the feasibility of the SSB method comprehensively.

## 6 Conclusions

Performance evaluation of a TLS is a critical concern for ensuring the quality of measurements in engineering applications. Measuring a long scale bar is one way to realize the length tests described in the ASTM E3125-17 standard. Considering the challenges in producing a stable long-length artifact, we proposed the SSB method, where a short scale bar is stitched together to provide spatial length reference of several times its length. We demonstrated the validity of this technique through both model-based and experimental analysis and then showed its application in realizing the symmetric length tests described in the ASTM E3125-17 standard. The clear advantages of a short scale bar are that it can be calibrated in a laboratory, it is portable, and it is more stable over time than a long scale bar. The disadvantage of the SSB method is that the uncertainty in the stitching process increases linearly with increasing number of segments used; this is not necessarily a drawback for TLSs with one or two segments in the stitching process, because the accuracy specifications are generally very large in comparison to the uncertainty in the stitched reference length.
